# Exploring Loneliness, Fear and Depression among Older Adults during the COVID-19 Era: A Cross-Sectional Study in Greek Provincial Towns

**DOI:** 10.3390/healthcare11091234

**Published:** 2023-04-26

**Authors:** Lamprini Moustakopoulou, Theodoula Adamakidou, Sotirios Plakas, Marianna Drakopoulou, Paraskevi Apostolara, Alexandra Mantoudi, Dimos Mastrogiannis, Afroditi Zartaloudi, Stelios Parissopoulos, Alexandra Koreli, Marianna Mantzorou

**Affiliations:** MSc Program in Community and Public Health Nursing, Nursing Department, School of Health and Care Sciences, University of West Attica, 12243 Athens, Greece; kn20009@uniwa.gr (L.M.); thadam@uniwa.gr (T.A.); skplakas@uniwa.gr (S.P.); mdrakopoulou@uniwa.gr (M.D.); vapostolara@uniwa.gr (P.A.); amantoudi@uniwa.gr (A.M.); dmastrogiannis@uth.gr (D.M.); azarta@uniwa.gr (A.Z.); spariss@uniwa.gr (S.P.); akoreli@uniwa.gr (A.K.)

**Keywords:** COVID-19 pandemic, depression, fear, loneliness, older adults

## Abstract

Background: With the onset of the COVID-19 pandemic, life changed abruptly for older adults in Greece. Social isolation, lockdowns, the fear of serious illness and death, all contributed to an increased risk of developing depression. Objective: To explore the presence and severity of depression in older adults in Greek provincial towns during the pandemic and to explore any possible relationships with loneliness and fear. Methods: A convenience sample of 200 participants aged >65, completed a socio-demographic form, the Revised UCLA Loneliness Scale, the Geriatric Depression-15 Scale (GDS-15) and the COVID-19 Fear Scale (FCV-19S). Data were collected from April to May 2022. Results: The average age of participants was 76.6 years and 35.5% of the participants presented depressive symptoms (mean prevalenceof depression 5 ± 3.7). A moderate to low level of loneliness (mean value 39 ± 11.3) and a moderate level of COVID-19 fear (mean value 18.9 ± 6.5) were also experienced. Higher levels of loneliness were found among participants with lower perceived health status and among those participants registered in Primary Health and Social Care (PHSC) services. Loneliness was positively correlated with depression (r = 0.7, *p* < 0.001), and increased loneliness and depression were associated with an increase in fear of COVID-19 (r = 0.2, *p* = 0.01 for both). Conclusions: During the pandemic, older adults experienced loneliness, fear of COVID-19 and depression which were positively associated with each other. It is imperative to develop PHSC policies that are aimed at addressing the mental health problems of the older population, which have been caused by the COVID-19 pandemic, through developing their resilience, offering psychological support and promoting social connections.

## 1. Introduction

The COVID-19 pandemic has been an uncontrollable and stressful event. Measures to limit the transmission of the virus, such as curfews and home confinement as well as the unparalleled number of deaths, seem to have had an impact on the well-being and mental health of the entire population [1,2,3,4].

The sudden appearance of the COVID-19 pandemic did not leave the mental health of older adults unaffected. The COVID-19 pandemic seems to have led to significantly increased levels of anxiety and depression even in persons with no previous history of mental illnesses [5]. Furthermore, due to home confinement and the strong dependence of older adults on caregivers, there are concerns regarding increased cases of psychological and physical abuse among the older population [6].

Fear has been one of the most often experienced emotions during the COVID-19 pandemic [7]. The predisposing factors for fear are high rates of infection, anxiety, uncertainty, risk of infection and death of loved ones and media exposure [8,9]. Due to the high and fast transmission rate and unexpected deaths, people have experienced increased levels of fear of COVID-19 which have affected the cognitive, physical and social functioning of older adults [10].

During the COVID-19 pandemic, people experienced extremely high levels of psychological distress which was occasionally clinically significant. Loneliness was another consequence of the pandemic. Loneliness can often carry a stigma, and unfortunately, it is often ignored despite being strongly associated with physical and mental morbidity and premature mortality [11]. People experienced a lack of personal control over social interactions [12]. Social isolation is a significant predisposing factor for poor mental health which has been operationalized as anxiety caused by the coronavirus [13]. Inevitably, social isolation enhances the feeling of loneliness. Social isolation refers to an objective state of restricted social relationships and contacts, while loneliness refers to a perceived feeling of social isolation. Social isolation and loneliness are important public health risks for older adults but are not adequately addressed [14].

The COVID-19 pandemic has led to a significant increase in anxiety and depression, especially in older adults [1,2,11]. Depression is a type of mood disorder and is ranked as the most prevalent mental disorder among older adults. Older adults more frequently report physical symptoms such as sleep disorders, decreased appetite and a strong feeling of fatigue [15]. Poor physical health tends to aggravate the onset of the disease in older adults [16]. Previous research findings have demonstrated that socially isolated older adults were at greater risk of developing depression and anxiety [17,18,19]. Under pandemic conditions and social exclusion, older adults are usually particularly vulnerable to suicide as they experience a strong feeling of disconnection from society, distancing and loss of socialization [20].

It is important to study the effects of the pandemic on the mental health of older adults, as the consequences of the pandemic may affect the right and ability for healthy aging and may lead to functional and cognitive decline, disability and even increased mortality [18,21,22,23]. The existing research data on the effects of the pandemic on the mental health status of older adults have shown a wide range in the prevalence of depressive symptoms which could be attributed to different cultural backgrounds, socioeconomic circumstances as well as different restrictive measures during the pandemic in each country. In Greece, research data are limited concerning the relationship between loneliness and depression of older adults during the COVID-19 pandemic, showing however a strong relationship between them; [24]; in addition, most studies have concerned age groups other than older adults [25,26].

## 2. Objective

The purpose of this study was to explore the presence and levels of depression in community-dwelling older adults in provincial towns of Greece during the pandemic and to explore any possible relationships with loneliness and fear. Secondary aims were to find possible correlations between socio-demographic characteristics and fear, loneliness and depression, and to compare the mental health of older adults who were were attending and were not attending Primary Health and Social Care (PHSC) services.

## 3. Materials and Methods

### 3.1. Study Materials

This community-based, cross-sectional, study of older adults was conducted in various Primary Health and Social Care (PHSC) services (home care, day care center for older adults (KIFI), walk-in day center for older adults (KAPI)) in four provincial towns in Greece. The data collection was performed during April–May 2022. Although, in Greece, community centers for older adults remained closed for a long period of the pandemic, during the study period, only vaccinated persons who were wearing a surgical mask could visit public services. Walk-in day centers only accepted a small number of older adults who were vaccinated or showed a negative rapid test. No recreational activities were available during this period, only medical prescriptions and physiotherapy sessions. Day care centers accepted a very restricted number of older adults, depending on the size of the premises. Home care services were obliged to diminish the frequency and length of visits. The final sample size was 200 participants. According to the G-power program, 200 was a sufficient number of participants for a correlation study in which a multivariate linear regression analysis could be performed with a power of 95%, an error level of α = 0.05, an effect size of at least 0.15 and a total number of controlled predictors of 5.

The sample consisted of participants over the age of 65 who had the cognitive, physical and speech ability to understand and complete the questionnaires. The participants were divided equally into two groups: (a) those who were registered at PHSC programs before the pandemic and were conveniently selected and (b) those who had never been registered at PHSC programs and were selected using snowball sampling techniques. Older adults who had been diagnosed with depression or dementia as well as older adults who had impaired physical and psychological functioning that hindered communication were excluded from participation in the study.

### 3.2. Data Collection

The first author and main researcher personally distributed the questionnaires to the participants in a quiet and private area of the facilities. Regarding the participants who were not registered in a PHSC program, they completed the questionnaires in their homes or through a telephone survey. Among the 230 eligible participants who were recruited, 200 participants finally completed the questionnaires; thus, the response rate was 87%.

### 3.3. Instruments

Data were collected by using a socio-demographic form and three questionnaires after permission was granted by the constructors of the questionnaires. The socio-demographic data collected were basic socio-demographic characteristics of the participants, such as gender, age, educational level, profession in the past, health status, participation in community programs, self-care ability, dealing with problems alone or with the help of family/friends/PHSC services, etc.

Loneliness was measured using the Revised UCLA Loneliness Scale [27], which is a 20-item self-report scale that describes feelings or thoughts about relationships. Questions that are positively expressed reflect satisfaction with interpersonal relationships (e.g., a sense of belonging to a group of friends). Negatively expressed questions reflect dissatisfaction with interpersonal relationships (e.g., a sense of lack of companionship). Responses are rated from 1 to 4 (1 = never, 2 = rare, 3 = sometimes, 4 = often). The lowest overall score is 20 and the highest overall score is 80. Higher scores indicate a greater sense of loneliness. The Greek version has been validated [28,29] and presented good internal consistency (α = 0.87–0.89).

Depression was measured using the Geriatric Depression-15 Scale (GDS-15) [30], which is a short screening tool that is specifically designed for self-assessment of depression in geriatric populations. Respondents were asked to answer 15 closed-ended questions with the responses yes or no. The Greek version of the GDS-15 has been validated [31] and showed high internal consistency with a Cronbach’s alpha of 0.94. The severity of depression was categorized based on the Greek validation of the Geriatric Depression Scale according to which 0–5 points corresponded to “absence of depressive symptoms”, 6–10 points corresponded to “moderate depression” and 11–15 points corresponded to “severe depression” [31].

Finally, fear was measured using the COVID-19 Fear Scale (FCV-19S) [32], which was designed to assess the level of fear associated with SARS-CoV-2. It includes seven (7) questions-statements that evaluate the fear of COVID-19. It is a self-report instrument that is rated on a 5-point Likert scale with 1 corresponding to “I absolutely disagree” and 5 corresponding to “I completely agree”. Overall scores could range from 7 to 35, with higher scores representing greater fear. The Greek version has been validated by Tsipropoulou et al. (2021) and presented good internal consistency (α = 0.87) [33].

### 3.4. Ethical Approval

After the participants were informed about the purposes and benefits of the study, they were assured that all personal data would remain confidential and that they were free to withdraw at any stage of the study. Then, they signed a written consent and complaint form. Permission was obtained by the scientific boards of the municipalities of the Primary Health and Social Care Services in which the study was conducted, and then permission was granted by the Ethics Committee of the University of West Attica (protocol number 4136, 26 January 2022).

### 3.5. Data Analysis

Absolute and relative frequencies were used to present categorical variables; mean, standard deviation, median, minimum value and maximum value were used to present quantitative variables. The Kolmogorov–Smirnov test was used and the quantitative variables showed a normal distribution. Associations among loneliness, depression, and fear of COVID-19 were investigated using the Pearson correlation coefficient. The independent variables in the present study were the demographic characteristics of the participants and the dependent variables were loneliness, depression and fear of COVID-19. The *t*-test, the Pearson correlation coefficient, the Spearman’s correlation coefficient and multiple linear regression analysis were used in the data analysis. To investigate the relationship between two quantitative variables, Pearson’s correlation coefficient was used. Spearman’s correlation coefficient was used to investigate the relationship between a quantitative variable and an ordinal variable. Associations among loneliness, depression and fear of COVID-19 were explored using Pearson’s correlation coefficient.The data analysis was conducted with the IBM SPSS 21.0 software and the significance level was set at *p* < 0.05.

## 4. Results

### 4.1. Demographic Characteristics

The demographic profile of the 200 participants is presented in Table 1. The mean age of the participants was 76.6 years. Most participants were women (59.5%), primary school graduates (49%) and had children (95%). Most participants lived with others (73%) and stated that they took care of themselves on their own (78%); 51.5% stated that their health status was moderate. Half of the participants were enrolled in a PHSC program, among which 50% of the participants were enrolled in home care; 25% of the participants were walk-in day center enrollees; 25% of the participants were day care enrollees. Most participants said that they faced their problems with the support of their family (71%).

### 4.2. Fear of COVID-19

The COVID-19 Fear Scale (FCV-19S) presented a Cronbach’s alpha of 0.92 which indicated high reliability of the questionnaire in this study.

The older adults’ scores on the FCV-19S are shown in Table 2. It should be emphasized that an increase in the score indicates an increase in fear of COVID-19. The mean fear of COVID-19 score was 18.9, which indicated a moderate level of fear of COVID-19. The bivariate analysis revealed a statistical relationship at the level of 0.20 (*p* < 0.20) between five independent variables and the fear of COVID-19. Then, the multivariate linear regression was applied. However, no statistically significant relationship was found.

### 4.3. Loneliness: The UCLA Loneliness Scale Presented a Cronbach’s Alpha of 0.9 Which Indicated High Reliability of the Questionnaires in This Study

The older adults’ scores on the UCLA Loneliness Scale are shown in Table 2. The mean loneliness score was 39, which indicated a moderate to low level of loneliness experienced by participants. The bivariate analysis resulted in a statistical relationship at the level of 0.20 (*p* < 0.20) between eight independent variables and loneliness. For this reason, a multivariate linear regression was applied, and the results are presented in Table 3. In the regression model, all independent variables (enter method) were entered at the same time and their statistical significance was estimated.

The value for the Durbin–Watson statistic was 2, which indicated the independence of the observations. The value for ANOVA was <0.001 which indicated the statistically significant effect of independent variables on the dependent variable. The tolerance and VIF values were acceptable for all independent variables. Acceptable tolerance values are >0.5, and acceptable VIF values are <4. According to the results of the multivariate linear regression, participants with worse self-perceived health status experienced greater loneliness. In addition, participants who participated in home care/day care services, who were usually persons with diminished self-care ability, experienced greater loneliness than those who did not participate in PHSC programs or attended walk-in care centers.

### 4.4. Depression

The Geriatric Depression Scale (GDS-15) presented a Cronbach’s alpha of 0.84, which indicated high reliability of the questionnaire in this study. The older adults’ scores on the GDS-15 are shown in Table 2. An increase in the GDS-15 score indicated an increase in the depression experienced by the participants. The mean depression score was five. According to the categorization of depression severity, 66.5% of the participants (*n* = 133) had no depressive symptoms, 22.5% of the participants (*n* = 45) had moderate depression and 11% of the participants (*n* = 22) had severe depression. The bivariate analysis resulted in a statistical relationship at the level of 0.20 (*p* < 0.20) between nine independent variables and depression. Thereupon, a multivariate linear regression was applied, and the results are presented in Table 4. In the regression model, all independent variables (enter method) were entered at the same time and their statistical significance was estimated.

The value for the Durbin–Watson statistic was 1.9, which indicated the independence of the observations, since values around 2 indicate the independence of the observations. The value for ANOVA was <0.001 which indicated the statistically significant effect of independent variables on the dependent variable. The tolerance and VIF values were acceptable for all independent variables. According to the results of multivariate linear regression, participants with worse self-perceived health status experienced greater depression, and those who needed the help of others to take care of themselves experienced greater depression than those who could take care of themselves.

### 4.5. Correlation of Loneliness, Depression and Fear of COVID-19

The correlations among loneliness, depression and fear of COVID-19 are shown in Table 5. The statistically significant relationships found were: (1) an increase in loneliness was associated with an increase in depression and an increase in fear of COVID-19 and (2) an increase in depression was associated with an increase in fear of COVID-19.

## 5. Discussion

The purpose of the present study was to explore the presence and severity of depression during the COVID-19 pandemic and to determine possible correlations with loneliness and fear of COVID-19 among older adults. The study results revealed a mean value for depression severity of 5 ± 3.7, with 33.5% of participants experiencing depressive symptoms. Previous studies conducted before the pandemic on depression prevalence of older adults in Greece have shown that the prevalence of depressive symtoms varied according to the geographical areas (30.2%, 45.2% and 46.2%, respectively) [34,35,36]. Moreover, studies conducted during the first year of the pandemic around the world also presented a wide range of percentages for the prevalence of depressive symptoms. In Poland, 26.25% of older adults presented depressive symptoms [37]; in Canada, 26.4% of older adults presented depressive symptoms [38]; in Turkey, 37.5% of older adults presented depressive symptoms [39]; in Jordan, 37.1% of older adults presented depressive symptoms [40]; in China, the prevalence of depression reached 37% [41] and 47.5% [42]. Lower depression prevalence (15%) was found in India which could be attributed to higher resilience, and strong support by family due to their culture [43]. The differences in depression prevalence compared to the current study were probably due to the different study periods, since the studies were conducted in the first year of the pandemic when death rates were higher and vaccines had not yet been constructed. Moreover, China was the first country where COVID-19 appeared. In addition, different preventive measures were taken in each country, and different tools were used in the studies.

In the post lockdown period, a study in Israel showed that 40.4% of the participants presented mild depressive symptoms, while 10.8% of the participants presented moderate to severe symptoms [44]. These results, as well as the results of the current study which also took place after the cessation of the Greek lockdown, indicate that depressive symptoms persist as older adults face challenging adaptation difficulties while returning to their normal pre COVID routine of life [44]. These findings also present a significant challenge for health professionals.

Participants in the current study showed a moderate fear of COVID-19 (mean 18.9 ± 6.5) and a moderate to low level of loneliness (mean 39 ± 11,3). Positive correlations were found among depression, fear and loneliness. It must be pointed out that the research was conducted 27 months after the onset of the COVID-19 pandemic. This means that the study did not record the peak of the psychological effects of the pandemic on older adults, but only some of the effects. In addition, among older adults, mortality from COVID-19 decreased in the given time period, since 20,904,597 vaccinations had already been administered in Greece.

The prevalence of depressive symptoms in the sample of the present study is noteworthy, since 66.5% of participants experienced no depressive symptoms, 22.5% of participants experienced moderate symptoms and 11% of participants (*N* = 22) experienced severe depression. A special focus should be placed on the 11% of the sample who experienced severe and undiagnosed depression. Since diagnosed depression was an exclusion criterion from the present study, it becomes evident that this percentage is significant. Given that the mental health of older adults is particularly vulnerable in pandemic conditions and that there is a risk of suicidal ideation, the 11% of undiagnosed and severe depression is a serious finding that should be further evaluated. Of course, this percentage could be attributed to other pre-COVID existing factors such as the low economic status of pensioners in Greece as well as the social and welfare conditions regarding the care of older adults.

Older adults enrolled in Primary Health and Social Care (PHSC) facilities experienced higher levels of depression, fear and loneliness. More specifically, the mean depression score of those not enrolled in PHSC programs was 4, while the mean score of those enrolled in home care programs was 6.4 and of those enrolled in KIFI was 7.3. These results could be attributed to the fact that older adults who are not enrolled in PHSC programs usually have a better health status, are more active in their daily lives and have already developed their own family and social network, as a result of which they cover their emotional and social needs.

Higher levels of depression were experienced by older adults in poorer self-perceived health status and older adults who needed help from others for their care (mean 8.2) than those who could take care of themselves. Older adults in poor health tend to isolate themselves and develop feelings of loneliness. Additionally, older adults who have difficulty in developing a connection with their social network are more vulnerable to the development of depression and anxiety [2], which is also confirmed by the results of our research. Furthermore, quarantine per se is a strong predictor of the development of depression [45] and is thought to particularly affect those older adults who are most vulnerable due to ill health and need of help with their care. Finally, the social isolation imposed during a pandemic predicts greater chances of depressive symptoms [13].

In a related study by Parlapani et al. (2020) in Greece, 81.6% of the sample presented moderate to severe depressive symptoms, especially in women [24]. This high percentage may be due to the fact that this study was conducted three weeks after the imposition of a national lockdown in Greece. In addition, it is believed that these findings may also be related to the fact that, due to Greek culture, older adults are mostly responsible for taking care of their grandchildren and social distancing deprived them of this role. In addition, there was a strong feeling of fear due to deficiencies in the National Health Systems worldwide which were facing important shortages of healthcare professionals, increasing rates of patients and a lack of protective equipment, which led to a high levels of anxiety and depression among older adults [14,24]. Τhe present study and the study by Parlapani et al. presented different levels of depressive symptoms because they were conducted in different periods of the pandemic. The present study was conducted 27 months after the onset of the COVID-19 pandemic, while the study by Parlapani et al. [24] was carried out at the beginning of the pandemic. Moreover, our study was conducted in Greek provincial towns where the consequences of the quarantine tended to be milder.

Regarding the fear of COVID-19, the present study showed moderate levels of fear with a mean value of 18.9. Although mortality rates from COVID-19 in the study period were no longer as high as in the beginning of the pandemic, there was still a presence of fear among older adults as the pandemic was far from coming to an end. According to Mertens et al. (2020), the main factor of COVID-19 fear is fear for our loved ones [8]. It is likely that older adults were still feeling fear about COVID-19 as there was still a chance that they and their loved ones could get sick. Some researchers have concluded that older adults were more compliant with preventive measures [46]. It is believed that this was the case in order to protect both themselves and their loved ones. In addition, when thinking about the possibility of contracting COVID-19, they inevitably feared the possibility of becoming a burden on their family as they would not be able to take care of themselves. Additionally, due to mutations, there is still the fear of a new wave of the COVID-19 pandemic. Finally, it is known that it is necessary for older adults, as a vulnerable group, to have another booster dose of vaccine. It is possible that some older adults are experiencing post-traumatic syndrome disorder (PTSD) from the COVID-19 pandemic [47]. A study by Palgi et al. (2021) found that vaccine hesitancy was associated with higher levels of post-traumatic stress in older adults [48]. PTSD symptoms provoked by the COVID-19 pandemic may increase the fear of COVID-19, constituting a bidirectional relationship [49].

Other studies in the international and Greek literature have reported similar results in terms of fear. It has been found that older adults during the pandemic experienced moderate levels of fear, with female gender being one the most important predictor [10,24,50,51]. However, in contrast to the above studies, the present study showed no statistically significant relationship between fear with any of the socio-demographic factors.

In this study, the level of loneliness was also found to be moderate with a mean score of 39. Similar levels of loneliness have been found in Jordan, the Middle East (41.4%) [40] and Canada (44.7%) [52]. Higher levels of loneliness were presented by participants of the study who had a poorer self-perception of own health and needed the help of others for their care. In many studies, positive perceptions of health status have been associated with fewer feelings of loneliness and less depression [40,53,54].

Furthermore, high percentages of loneliness were presented by older adults who managed their problems with the help of others. Physical health problems hinder social interaction and lead to social isolation [55]. Older adults with poor health inevitably need the help of others to care for them. Unfortunately, the pandemic affected this possibility due to the preventive measures which were stricter for older adults, since they were more vulnerable. The frequency of visits of friends and relatives decreased significantly, resulting in older adults experiencing greater loneliness, both emotional and social. Savage et al.’s study, in 2021, reported similar results, according to which higher levels of loneliness were experienced by older adults in moderate to poor health, older adults who lived alone, and older adults who needed help with their care, where 43.1% stated that they felt loneliness sometimes and 8.3% felt loneliness often or always [52]. The study by Alhalaseh et al. (2022) also showed that loneliness was significantly associated with being functionally dependent and having poor self-perceived health as well as being concerned about contracting the COVID infection [40].

In addition, the present study showed higher levels of loneliness among older adults enrolled in the home care and daily care programs (KIFI). This fact may be due to the nature of the programs, as they are aimed at older adults who are experiencing loneliness, and therefore, it is likely that the percentages of loneliness would be the same if the research was conducted before the pandemic. As previously mentioned, the structures of KIFI and KAPI during the long period of the pandemic remained closed based on the relevant decree and, during the study period, a restricted number of older adults could access them but no recreational activities were offered. The KIFI and KAPI programs provide a way out for registered members to have the opportunity to socialize and entertain themselves through various activities on a daily basis.

The seniors’ participation in social networks and programs that offer recreational activities is believed to offer them a sense of security and, in many cases, a sense of “belonging” [56]. The inability to attend the recreational activities organized by the PHSC services during the pandemic period deprived them of beneficial social interaction and had inevitable effects on their mental well-being. This fact resulted in strengthening the feeling of lack of interaction and communication both with their peers and with the staff with whom they had developed strong ties. Therefore, the feeling of loneliness is inevitably intensified among the registered members, especially among thοse who live alone. In addition, during the pandemic, the “home care” programs throughout Greece were called upon to cover the needs of all citizens who were in need and not only those regularly served. This fact, combined with the strict instructions and recommendations of the relevant services for protection against the COVID-19 pandemic, inevitably affected the frequency and length of visits as well as the provision and quality of services to the beneficiaries of these programs.

In their study, Parlapani et al. (2020) found higher levels of loneliness among adults aged ≤80 years and among adults living alone [24]. Studies have shown that women were more affected in terms of loneliness by COVID-19 as they usually have a larger social network than men and choose more frequent face-to-face meetings. Gender has been considered to be a predictor of loneliness [57]. However, the present study did not find a statistically significant relationship between loneliness and gender.

In the present study, statistically significant correlations were found among fear, loneliness and depression. Since the beginning of the pandemic, older adults have been considered to be a vulnerable group. High mortality rates from COVID-19 among older adults and preventive measures were stressors that increased feelings of fear, loneliness, and the presence of depression [1,2,3,4,5]. According to the present study, higher levels of loneliness and depression prevalence were presented by older adults with poorer health and who were considered to be particularly vulnerable to COVID-19.

The fear of illness leads to social isolation and this results in an increase in the level of loneliness. The largest percentage of the group of the study is quite old, and COVID-19, similar to any other severe disease, poses a threat to them and is associated with death along with the accompanying pervasive fear and death anxiety [58]. Fear can cause psychiatric disorders such as post-traumatic stress disorders, anxiety disorders and depressive disorders [51]. Increased psychological problems such as depression are factors associated with fear of death [59].

Similar to the current study, Parlapani et al. found a positive correlation between loneliness and depression. They concluded that depression contributed to loneliness, since people with depression tended to isolate themselves [24]. Additionally, many longitudinal as well as cross-sectional studies have observed strong relationships between loneliness and depression. Beutel et al. (2017) found that greater loneliness in the general population was associated with higher prevalences of clinically significant depression, anxiety and suicidal ideation, regardless of age, gender and socioeconomic status [60]. During the pandemic, a significant correlation between the level of loneliness and the prevalence of depressive symptoms was also found in Dziedzic et al.’s study (2021) [37] and Alhalaseh et al.’s study (2022) [40]. Social isolation is a significant predisposing factor of poor mental health [13].

Protecting older adult’s mental well-being and screening should be a public health priority, since the consequences of poor mental health can be devastating [61,62]. Strengthening resilience of older adults through multiple interventions and by offering them psychological support through innovative technology-based interventions such as telematic monitoring programs are some approaches that could be implemented [63] as long as they consider the personal fears, values and priorities of each culture [64]. Mitigation of exposure to misinformation is yet another protective factor of safeguarding the mental well-being of the geriatric population. Promoting online social connections using communication platforms or various social media may contribute to healthy aging in the post COVID-19 era and beyond [65]. The mental health of the geriatric population has been negatively affected worldwide and it is imperative to develop primary health care policies that not only prevent and mitigate but also manage the mental health problems of the geriatric population caused by the COVID-19 pandemic.

### Study Limitations

This study is a cross-sectional study and, by definition, its results reflect a specific moment in time, the moment of measurement for each participant [66]. This fact results in the inability to extract reliable results regarding the causal relationship between the variables. Moreover, it is possible that the results regarding health status are not representative and reliable as no questionnaire was used to assess the health status, and relative information was provided based on the subjective judgment of each participant. Moreover, no cognitive tests were carried out to rule out dementia in the non-registered respondents for whom no medical file could be examined. In addition, despite the fact that subjects diagnosed with depression were excluded, the possibility that this study reflects pre-existing depressive symptoms in some cases cannot be ruled out. In conclusion, it is recommended that future studies should be prospective, use more research tools and, ideally, should use mixed methodologies in order to elicit better results.

## 6. Conclusions

The COVID-19 pandemic has contributed to many changes in older adults’ lives. The physical and mental health of the geriatric population, as expected, have been adversely affected. The pandemic has already lasted for three years. Death rates remain high in several countries, as well as infection levels, media overexposure and misinformation add to the feeling of uncertainty and intense concern. Inevitably, there is increased fear of COVID-19. Measures to contain the COVID-19 pandemic, such as physical distancing, have increased the level of social isolation.

Based on the results of the present study, it is evident that the COVID-19 pandemic has affected the mental health of older adults in terms of fear of COVID-19, loneliness, and depression. Among the notable results of the study are the statistically significant relationships found among depression, loneliness and fear of COVID-19, as well as the fact that 11% of undiagnosed severe depression cases were detected. The users of the services of the PHSC programs also presented higher levels of loneliness compared to the participants who were not users of these services, since the pandemic affected their ability to socialize and participate in the recreational activities that are offered by these services.

## Figures and Tables

**Table 1:** Demographic characteristics of the participants.

Demographic Characteristics	Ν	%
**Gender**		
Male	81	40.5
Female	119	59.5
**Age ^α^**	76.6	7.6
**Education level**		
Illiterate	27	13.5
Primary school	98	49
Secondary school	27	13.5
High school	23	11.5
Technological Educational Institution (TEI)	9	4.5
University	12	6
Master’s/Beyond Master’s	4	2
**Past occupation**		
Unskilled worker	8	4
Skilled worker	15	7.5
Freelancer	32	16
Farmer	45	22.5
Civil servant	30	15
Private employee	19	9.8
Housework	51	25.5
**Number of children**		
0	10	5
1	23	11.5
2	99	49.5
3	45	22.5
>3	23	11.5
**Living with others**		
No	54	27
Yes	146	73
**Number of family members that live together**		
0	54	27
1	84	42
2	39	19.5
3	10	5
>3	13	6.5
**Self-perceived Health status**		
Good	77	38.5
Moderate	103	51.5
Bad	20	10
**Self-care**		
Alone	156	78
Often with the help of others	29	14.5
Always with the help of others	15	7.5
Enrollee in PHSC program		
Home care	50	25
Walk in Day Center for older adults (KAPI)	25	12.5
Day Care Center for older adults (KIFI)	25	12.5
None	100	50
**Facing problems**		
Alone	21	10.5
With the support of family	142	71
With the support of friends	5	2.5
With the support of PHSC programs	32	16

^α^ mean, standard deviation.

**Table 2:** Scores of older adults on the FCV-19S, the UCLA Loneliness Scale and the GDS-15.

	Mean	SD	Median	Minimum	Maximum	Range of Score
FCV-19S	18.9	6.5	19	7	35	7–35
UCLA Loneliness Scale	39	11.3	37	23	73	20–80
GDS-15	5	3.7	4	0	14	0–15

**Table 3:** Multivariate linear regression with loneliness as the dependent variable.

Independent Variable	Coefficient b	Confidence Interval 95% of b	*p*-Value	Collinearity Diagnostics	
				Tolerance	VIF
Self-perceived health status	−7.1	−9.3–−4.8	<0.001	0.67	1.48
Home care/day care in relation to the absence of PHSC program/walk-in day center	3.5	0.3–6.7	0.03	0.68	1.46
Facing problems with the support of PHSC programs in relation to support of family/friends	3.6	0.3–7	0.03	0.65	1.54
Educational level	−0.02	−1.09–1.04	0.97	0.80	1.26
Number of children	−0.67	−1.87–0.52	0.26	0.81	1.23
Living with others	1.98	−6.06–2.10	0.34	0.58	1.73
Number of family members that live together	−0.11	−1.62–1.40	0.88	0.51	1.95

**Table 4:** Multivariate linear regression with depression as the dependent variable.

Independent Variable	Coefficient b	Confidence Interval 95% of b	*p* Value	Collinearity Diagnostics	
				Tolerance	VIF
Self-perceived health status	−2	−2.8 to −1.3	<0.001	0.60	1.67
Caring with the help of others in relation to self-care capability	2.3	1.1 to 3.4	<0.001	0.61	1.65
Loneliness score	0.18	0.15 to 0.22	<0.001	0.71	1.42
Fear of COVID-19 score	0.04	−0.01 to 0.09	0.15	0.92	1.09
Age	−0.01	−0.06 to 0,04	0.77	0.74	1.36
Educational level	−0.17	−0.42 to 0.09	0.20	0.78	1.29
Number of family members that live together	−0.15	−0.42 to 0.11	0.26	0.91	1.11
Facing problems with the help of others	0.16	−0.67 to 0.98	0.71	0.66	1.52

**Table 5:** Correlations of loneliness, depression and fear of COVID-19.

	Loneliness	Depression
**Depression**	0.7 (<0.001)	
**Fear**	0.2 (0.01)	0.2 (0.01)

Values are expressed as a Pearson correlation coefficient (*p*-value).

## Data Availability

Datasets used and/or analyzed during the current study are available on reasonable request from the corresponding author. The data are not publicly available due to privacy reasons.
